# Uniqueness of Companion Animal Fecal Microbiota: Convergence Patterns Between Giant Pandas, Red Pandas, and Domesticated Animals

**DOI:** 10.3390/microorganisms14010112

**Published:** 2026-01-05

**Authors:** Shuting Liu, Hairong He, Han Han, Hong Zhou, Yuxiang Chen, Huawei Tian, Shibu Qubi, Minghua Chen, Yonggang Nie, Wei Wei

**Affiliations:** 1 Key Laboratory of Southwest China Wildlife Resources Conservation, Ministry of Education, China West Normal University, Nanchong 637009, China; shutiaoliu@126.com (S.L.); h19802844548@163.com (H.H.); hanghang739@hotmail.com (H.H.); zhouhong1026@163.com (H.Z.); chenyx9708@126.com (Y.C.); tianhwv587@163.com (H.T.); 2 Liziping Giant Panda’s Ecology and Conservation Observation and Research Station of Sichuan Province, Nanchong 637009, China; 3 Sichuan Meigu Dafengding National Nature Reserve Administration, Meigu 616457, China; meigudafengding@163.com (S.Q.); meigunet@163.com (M.C.); 4 Institute of Zoology, Chinese Academy of Sciences, Beijing 100101, China

**Keywords:** high-throughput sequencing, gaint panda, sympatric wildlife, fecal microbiotahigh

## Abstract

To investigate the influence of host ecological niche on fecal microbial community composition, this investigation employed high-throughput sequencing to characterize the microbiota composition in fecal samples. Giant pandas (GP), red pandas (RP), sympatric wildlife (SA), and domesticated animals (HA) in the Meigu Dafengding National Nature Reserve were used in the research. The research has found that GP bacteria are mainly composed of *Proteobacteria* and *Pseudomonas*, RP is enriched in *Proteobacteria* and *Arthrobacter*, SA is characterized by *Firmicutes* and *Bacillus*, and HA is dominated by *Firmicutes* and *UCG-005 (uncultured Lachnospiraceae)*. In terms of fungi, GP and RP are mainly dominated by *Ascomycota*, enriched in *Mrakia* and *Thelebolus*, respectively, while SA is dominated by *Ascomycota* and *Thelebolus*, and HA is dominated by *Chytridiomycota* and *Geotrichum*. The assessment of alpha diversity demonstrated that HA had the highest microbial diversity and GP had the lowest; evaluation of beta diversity established that the community structures of each group were significantly separated. The study revealed a significant ecological divergence between the bacterial and fungal communities in sympatric wildlife, while giant pandas exhibit synergistic variation in their microbiota. This study provides new scientific basis for wildlife conservation from the perspective of focusing on microbial interactions.

## 1. Introduction

A Chinese endemic, the giant panda (*Ailuropoda melanoleuca*) is a flagship species functioning as a classic umbrella species in its ecosystem. Due to its habitat mainly being primitive bamboo forests at an altitude of 1800–3200 m, the protected area can simultaneously maintain the habitats of a large number of animals in the same area while protecting giant pandas, forming a significant umbrella effect. Research has shown that over 96% of giant panda habitats overlap with other endemic amphibians, birds, and mammals, with common sympatric wildlife including red pandas, golden monkeys, antelopes, and water deer [1]. In addition, wild boars, Asian black bears, and domesticated cattle also appear in the same mountain range, forming resource sharing or competitive relationships [2]. These same domain species have varying degrees of ecological niche overlap with giant pandas in forest structure, bamboo resources, and altitude gradients. Protecting the habitat of giant pandas can enhance overall biodiversity, and improve the stability and restoration capacity of regional ecosystems [3]. However, this crucial umbrella effect and the complex species network it sustains are facing increasing threats such as human activities.

Nowadays, the habitats of giant pandas and their coexisting animals are being disturbed by human activities, especially their livestock grazing activity and food resources. These interferences have already had an impact on their microbial system. The introduction of livestock not only occupies the original ecological niches of these animals but also, through fecal deposition, directly introduces livestock-specific bacterial and fungal assemblages, thereby potentially altering the ambient microbial composition of the habitat. Comparative microbiome analyses in previous research have revealed a distinct divergence in fecal microbial communities between domestic and wild ungulates [4]. The colonization of such exogenous microorganisms may further disrupt soil microecology, potentially intervening in the rhizosphere environment of bamboo plants, and ultimately having a chain effect on the quality of food resources that giant pandas and their sympatric wildlife rely on for survival. Against the backdrop of increasingly fragmented habitats, the feeding strategies of wild animals tend to be limited, and their gut microbiota undergoes adaptive restructuring of structure and function [5]. Notably, a cross species microbial exchange network has been established between domesticated animals and wild animals. Recent metagenomics research has also identified a fusion microbial gene pool contributed by both domestic and wild animals in the surrounding environment of panda distribution areas [6].

Located in the Liangshan Yi Autonomous Prefecture, the Meigu Dafengding National Nature Reserve lies in the southwest of China’s Sichuan Province and is the core channel and population exchange hub for giant pandas in the region. As an important ecological corridor connecting isolated populations of giant pandas, its habitat quality and microecological security are particularly critical. The region is experiencing new ecological pressures brought about by the transformation from traditional livelihoods, especially the increasingly active livestock production activities, which have led to close contact between wild animals with overlapping household boundaries and free-range livestock [7]. The change in spatial pattern has created potential conditions for the diffusion and community integration of cross species microorganisms such as bacteria and fungi, posing a potential threat to the gut microbiota health of wild populations [8]. Therefore, a background investigation of wild animal feces was conducted in the protected area to systematically analyze the composition and diversity of bacteria and fungi in the feces, providing scientific basis for assessing animal health status and potential pathogen transmission risks, as well as formulating microbial level protection strategies.

The research on conservation biology based on the perspective of microecology provides new ideas for the protection of endangered species. This study focuses on giant pandas and their coexisting animals in the protected area, including wild animals such as red pandas and deer, as well as domestic animals such as cats and dogs. Conduct the following research: (1) systematically analyze the composition differences and distribution patterns of fecal microbiota among different animal groups; (2) analyze the differences in patterns between bacteria and fungi in different animal groups.

## 2. Materials and Methods

### 2.1. Research Location

**Figure 1:**
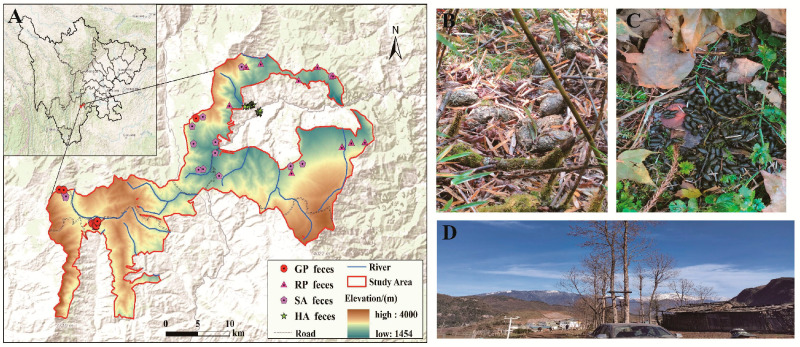
Sampling areas and distribution of sampling points.

The research area of this study is the Meigu Dafengding National Nature Reserve (Figure 1), with geographical coordinates of 102°52′–103°20′ E longitude and 28°30′–28°50′ N latitude [9]. The terrain belongs to the deep cut Zhongshan landform type, with a southwest to northeast slope, an altitude of 1356–4042 m, and a vertical height difference of 2686 m. The climate in the protected area is cold, humid, rainy, and foggy, and has a frost-free period of 230–280 days [10]. According to the 2021 National Key Protected Wildlife List, there are a total of 59 species of nationally protected wildlife in the protected area. Among them, there are 10 species of national first-class protected animals, including giant pandas and Sichuan snub-nosed monkeys, and there are 49 species of second-class protected animals, including red pandas, Tibetan macaques, red-bellied pheasants, and blood pheasants [11]. As the core gene corridor connecting the local populations of giant pandas in Mabian, Yuexi, and Leibo counties, census data from the Fourth National Giant Panda Survey documented a population of 186 wild pandas in the reserve, ranking it among the highest-density habitats nationally [12]. However, this important link hub is facing potential threats to the “lifeline” that maintains population health, as it is difficult to eliminate the entry of domestic dogs, poultry, and other animals into the forest area due to intensive human activities in the surrounding area.

### 2.2. Sample Collection

This study conducted animal feces sample collection in the reserve from December 2024 to June 2025. The collection effort yielded 108 stool samples, including 13 samples from giant pandas, 26 red pandas, 28 sympatric wildlife (including tufted deer, Malayan porcupine, Tibetan macaque, Asian hog badger, blood pheasant, lady Amherst’s pheasant), and 41 domesticated animals such as cats, dogs, chickens, ducks, goose, sheep, pig, cattle, horse (Table A1). From the surrounding village of Longwo Township, animals were categorized into four groups based on their ecological context and spatial relationship to the focal giant panda habitat: (1) GP: the focal species, giant panda; (2) RP: the red panda, serving as an ecological comparator sharing a similar forest and dietary niche; (3) SA: sympatric wild animals co-occurring within the same protected forest reserve; (4) HA: animals living in human settlements adjacent to the reserve. Prepare disposable sterile-sealed bags, label paper, 5 mL sterile centrifuge tubes, marker pens, portable GPS (to record sampling point coordinates), and mobile phones (to take photos to record the original state of feces and surrounding environment) before sampling; during the sampling process, wear disposable sterile PE gloves for operation, use a disposable sterile sampling spoon to excavate the internal feces, and immediately transfer them into a sterile disposable sealed bag, while indicating species information, sampling time, coordinates, altitude, and sampler. Freeze preservation: After returning to the laboratory, each original sample is divided into three pre-labeled 5 ml centrifuge tubes and immediately placed in a −80 °C ultra-low temperature freezer for freezing preservation. It is strictly prohibited to pick up feces directly by hand throughout the process to avoid sample contamination caused by human factors; new PE gloves and sample tubes should be replaced during each sampling.

### 2.3. Extraction, Amplification, and Sequencing of DNA

All collected samples were transported by dry ice to Mingke Biotechnology (Hangzhou, China) Co., Ltd.) for 16S and ITS sequencing. This study used pacbio revio sequencing to obtain 16S and ITS information of the microbiome in the samples. The specific workflow was as follows: The target 16S rRNA and ITS fragments obtained by PCR amplification underwent end repair, A-tailing, and ligation with PacBio SMRTbell adapters to construct circular libraries. The purified libraries were bound to polymerase, loaded onto a Revio SMRT Cell, and sequenced using Circular Consensus Sequencing (CCS) technology to directly generate high-quality HiFi reads. Raw data were processed with SMRT Link, followed by quality control, denoising, and chimera removal using the QIIME 2 pipeline, ultimately yielding amplicon sequence variants (ASVs). Taxonomic annotation was performed based on the SILVA and UNITE databases for subsequent analysis of microbial community diversity and structure.

### 2.4. Data Analysis

According to 16S rRNA and ITS sequencing data, quality control and feature table generation were performed using the QIIME2 platform [13]. In the R language environment, data import and management are carried out through the phylosseq package, and a community composition bar chart is drawn using the ggplot2 package [14]. Using the Microeco package in R language for LEfSe [15], analyze and screen for inter group differences in ASVs using the Kruskal–Wallis test [16]. Perform linear discriminant analysis (LDA) to determine the significantly enriched bacterial and fungal groups in each group based on LDA scores > 3.0 and *p* < 0.05. The results are presented in an evolutionary branching diagram. Alpha diversity analysis uses the phylosseq package to calculate the phylogenetic diversity (PDw_holey_tree) index and Shannon index [17]. Kruskal–Wallis test was performed using the vegan package to compare differences between multiple groups, and the results were displayed in a box plot using the ggplot2 package. Beta diversity is calculated by utilizing Bray–Curtis dissimilarity matrices in conjunction with non-metric multidimensional scaling (NMDS), which is performed using the vegan package. The statistical significance of inter-group community structure differences is evaluated using PERMANOVA, and the results are visualized using the ggplot2 package [18]. Use the vegan package to calculate the Pearson correlation between bacterial and fungal PD index and Shannon index, and perform the Mantel test to analyze the correlation between community structures. Conduct Procrustes analysis through the vegan package [19]. Analyze and evaluate the similarity between bacterial and fungal community structures, and present the analysis results in a bivariate plot using the ggplot2 package. The R(4.5) environment serves as the platform for all statistical computations.

## 3. Results

### 3.1. Composition of Fecal Microbiota at Different Levels

**Figure 2:**
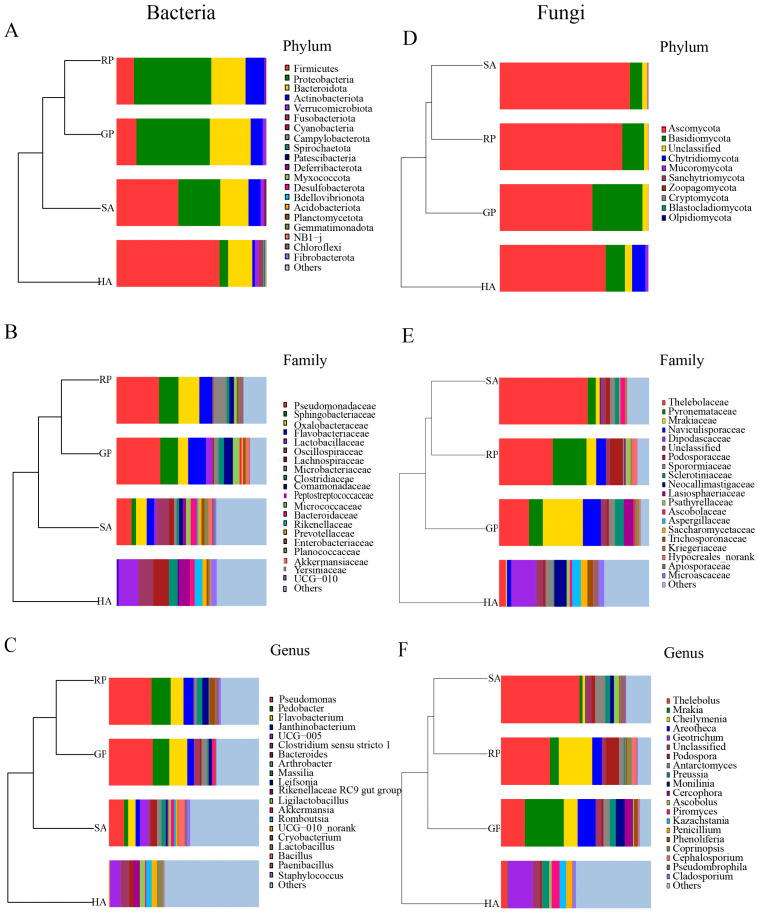
The differences in microbial community structure among different animal groups at multiple taxonomic levels. (**A**–**C**), respectively, demonstrate the composition of bacterial communities at the phylum, family, and genus levels. (**D**–**F**) displays the composition of fungal communities at the phylum, family, and genus levels. In each subgraph, different colors represent different classification units, and the height of the bar chart represents their relative abundance. Group abbreviation: GP, giant panda; RP, red panda; SA, sympatric wildlife; HA, domesticated animals.

At the bacterial phylum level (Figure 2A), the four groups of animals showed significant compositional differences. Both the GP giant panda group and the RP red panda group belong to the *Proteobacteria* and *Bacteroidota* for the phylum of dominant bacteria. A notable increase in the relative abundance of *Firmicutes* was observed within the SA group, while the HA domesticated animal group showed the highest diversity of phylum, among which *Spirochaetota* abundance was significantly higher than other groups. At the family level (Figure 2B), the community structures of GP and RP are most similar, with the dominant bacterial family being *Pseudomonadaceae*, with environmental-related bacterial families such as *Sphingobacteriaceae* [20]. Among them, GP specifically enriches the *Comamonadaceae* and the *Micrococcaceae*, and RP is unique to the *Microbacteriaceae*. In contrast, the microbiota of HA is dominated by the family *Oscillospiraceae,* characterized by *Lachnospiraceae* and *Lactobacillaceae*, and significantly enriched with *Prevotellaceae* and *Enterobacteriaceae*. Both SA and HA groups showed a higher proportion of *Bacteroidaceae*. However, the community structure of HA exhibits more typical characteristics of domesticated animals. At the genus level (Figure 2C), the inter-group differences were most significant. *Pseudomonas*’ high abundance was observed in the GP, RP, and SA groups. GP and RP groups specifically enriched the genus *Arthrobacter* compared to the genus *Leifsonia*. The proportion of *UCG-005(uncultured Lachnospiraceae)* in the SA group is also relatively high. In contrast, the microbial composition of the HA group is completely different, with the dominant genus being *Bacillus*, *UCG-005(uncultured Lachnospiraceae)*, and *Clostridium sensu stricto 1.*

The fungal community composition is also rich. According to Figure 2D–F, GP, RP, and SA samples have high similarity, while HA samples have significant differences from the first three. For *Ascomycota*, *Basidiomycota*, they are the dominant phylum of GP, RP, SA, and HA. Among them, the abundance of *Ascomycota* is relatively high in the feces of four animal species. For *Chytridiomycota*, it is highly abundant in HA. Compared with the first three groups of samples, the phylum composition of HA is more diverse, such as *Mucoromycota*, and the proportion increases in HA. At the family level, the fungal communities of GP, RP, and SA have a high similarity and are therefore clustered together, while HA has a lower similarity with the first three groups and forms a separate branch. The abundance of *Thelebolaceae* is relatively high in GP, RP, and SA. For *Dipodascaceae*, it is more abundant in HA. In addition, *Mrakiaceae* is observed from *Basidiomycota*, *Pyronemaceae*, and *Naviculisporaceae*. It is the dominant microbial community of GP, RP, and SA, while *Neocallistigaceae* and *Aspergillus* are the dominant microbial community of HA. At the level of the fungal genus, the Thelebolus from the *Ascomycota* is observed. Among GP, RP, and SA, *Mrakia* has the highest proportion (*Arcotheca* genus, the genus *Cheilymenia)*. Next up, among them, the abundance of *Arcotheca* was significantly greater in the GP group compared to the other three groups. On the contrary, the fungal community in the HA group is dominated by the *Geotrichum* genus, and mainly and significantly enriched in the *Piromyces* genus, Kazakhstan yeast genus, and the genus *Penicillium*.

### 3.2. Differences Between Fecal Microbiomes

**Figure 3:**
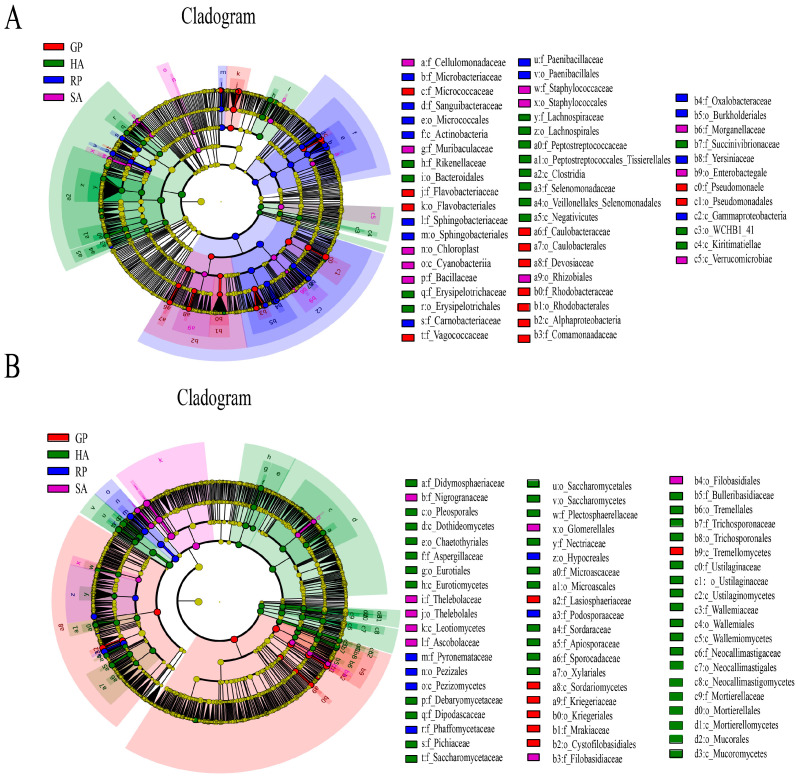
Cladogram of differentially abundant microbial taxa identified by LEfSe analysis. Circles from the inside out indicate the phylogenetic levels from the phylum to genus. (**A**) The red, green, blue, and purple circles represent the bacteria enriched in the sediment of GP, HA, RP, SA. (**B**) The red, green, blue, and purple circles represent the fungal enriched in the sediment of GP, HA, RP, SA. Respectively, whereas the yellow circles represent the taxa with no significant differences.

In the bacterial community (Figure 3A), each group presents different characteristic taxa: the GP giant panda group belongs to the *Micrococcaceae* and *Comamonadaceae* of the *Actinobacteria*. As a feature, the RP red panda group mainly accumulates *Paenibacillaceae*-like bacteria related to *Microbacteriaceae*, and the SA sympatric wildlife group is composed of *Staphylococcaceae* aureus. As a representative, the HA domesticated animal group showed the most diverse bacterial markers, with the most significant marker being the *Peptostreptococcaceae*. In the fungal community (Figure 3B), each group also showed unique marker groups: the fungal marker in the GP giant panda group was the *Kriegeriaceae* and the *Mrakiaceae*. *Pyronemaceae* in the RP red panda group was significantly enriched, and the characteristic fungus of the SA-associated animal group is *Thelebolaceae*, a member of the *Botrytis* family. The HA domesticated animal group is enriched in the family *Aspergillaceae* and *Apiosporace*.

### 3.3. Diversity of Fecal Microbiota

**Figure 4:**
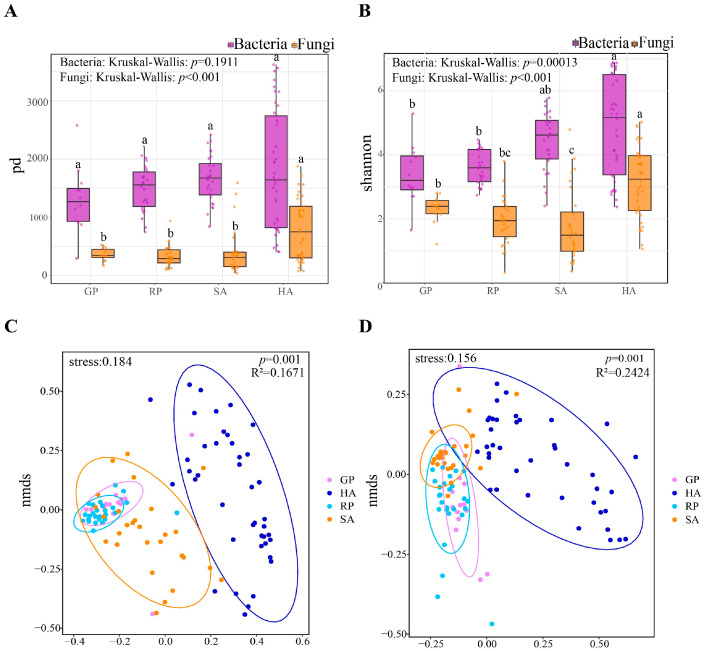
Inter-group comparison of fecal microbial community diversity: (**A**) PD index for bacteria and fungi; (**B**) Shannon index for bacteria and fungi; (**C**) NMDS analysis of bacterial community; (**D**) NMDS analysis of fungal community. Groups sharing a common letter are not significantly different, whereas groups labeled with distinct letters are statistically significant from each other.

In terms of the relationship between diversity-level and community structure, bacterial and fungal communities exhibit different patterns (Figure 4A–D). For bacterial communities, the HA domesticated animal group showed significantly higher alpha diversity than other groups, and their distribution in NMDS space was also significantly separated from other groups, indicating that domesticated animals not only have higher bacterial diversity, but also have unique community structures. In contrast, the GP giant panda group has the lowest alpha diversity in bacteria. The expression of fungal communities is more complex. The HA domesticated animal group has the highest fungal alpha diversity, followed by the GP giant panda group, which is different from the bacterial pattern (Table A2). Judging from the distribution of points (Figure 4C), the samples from each group are relatively separated in the two-dimensional space. In particular, GP and RP, as well as SA and HA, exhibit distinct clustering tendencies, indicating that the bacterial composition of each group possesses its own uniqueness. More importantly, NMDS analysis showed more significant inter group segregation of fungal communities (stress = 0.156), and the F-value of PERMANOVA analysis (11.0893) was significantly higher than that of bacteria (6.9567), indicating a stronger influence of host species on fungal community structure, which may be consistent with the inter-group differences observed in alpha diversity.

### 3.4. Intra-Group Similarity of Fecal Microbiota

**Figure 5:**
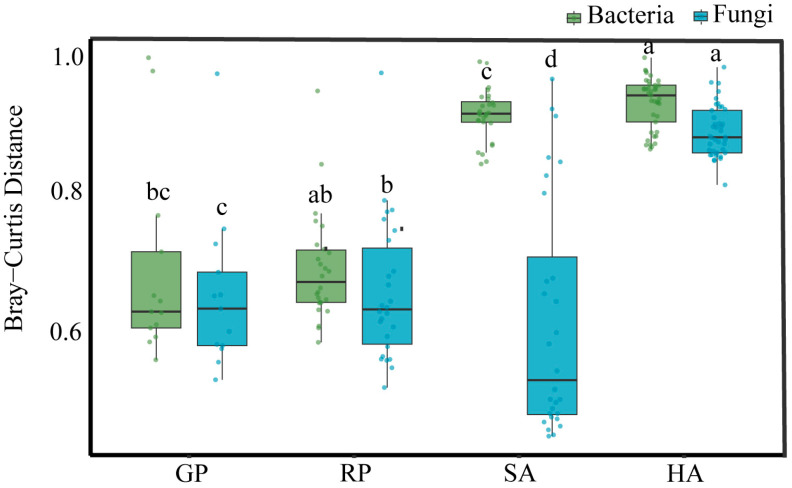
Analysis of within-group similarity based on Bray–Curtis distances. Groups sharing a common letter are not significantly different, whereas groups labeled with distinct letters are statistically significant from each other.

A comparison between bacterial and fungal community diversity in fecal microbiota of different animal groups (GP, RP, SA, HA) showed (Figure 5) that bacterial community diversity was higher than fungal community diversity in all groups (PERMANOVA, bacteria, R^2^ = 0.32, F = 12.85, *p* = 0.001; fungi, R^2^ = 0.48, F = 25.34, *p* = 0.001). However, the degree of difference between the two varies significantly depending on the group. Specifically, in the GP group, the diversity levels of bacteria and fungi are the closest, relative to the GP group, and the diversity of fungal communities in the RP and HA groups was lower than that of bacterial communities; particularly, in the SA group, the diversity of fungal communities was significantly lower than that of bacterial communities. The impact of different animals on fecal microbiota is selective [21,22,23,24], with the SA group being the most prominent.

In summary, our comparative analysis reveals distinct fecal microbial composition across the four animal groups. Giant pandas and red pandas, despite their phylogenetic distance, shared some convergent features. In contrast, sympatric wildlife and domesticated animals harbored more diverse and compositionally distinct microbiota.

## 4. Discussion

### 4.1. Bacterial Community Structure Exhibits Significant Inter-Group Variation

Bacterial community structures exhibited significant differences across the various animal groups. The *Comamonadaceae* family enriched in the GP giant panda group and *Micrococcaceae* all are typical soil and environmental bacteria [25,26,27,28]. This discovery supports the hypothesis that giant pandas ingest microorganisms from bamboo and the surrounding environment through feeding. It is worth noting that the *Comamonadaceae* has the ability to degrade complex organic compounds. The specific components in bamboo may be very important to the digestive process of giant pandas. *Paenibacillaceae* in the red panda RP population is significantly enriched, which can produce a variety of extracellular enzymes involved in cellulose and hemicellulose degradation. This discovery provides a new perspective for understanding the digestive adaptation mechanism of red pandas to plant food [29]. The specific enrichment of Staphylococcus in SA sympatric wildlife may indicate that these animals are in a specific physiological or environmental stress state [30]. This finding is consistent with the previous research results of evaluating the health status of wild animals [31], indicating that this population may be used as a potential biomarker for wildlife health monitoring. For *Peptostreptococcaceae* in the HA domestic animal group, this remarkable enrichment phenomenon is mainly attributed to the intake of high-protein feed or processed feed in captivity, which highlights the role of artificial feeding conditions in shaping intestinal microflora [32].

### 4.2. Fungal Community Characteristics Form a Distinct Ecological Pattern from Bacteria

The analysis results of fungal communities show completely different ecological characteristics from bacteria. The enrichment characteristics of *Mrakiaceae* in GP giant panda group are consistent with the environmental characteristics of high-altitude cool habitats [33]. This discovery provides important evidence for understanding the co-adaptation mechanism of the host fungi. It is particularly noteworthy that the cold-loving characteristics of *Mrakiaceae* may represent an evolutionary adaptation to the climate of a specific habitat, which may play the role of affecting the nutrient metabolism efficiency of the host. The *Pyronemaceae* in the red panda RP community are remarkably enriched, which reveals the close relationship between the fungal community and the forest ecosystem [28,33,34,35]. As a typical saprophytic bacteria, the role of this group in forest organic matter decomposition may indirectly affect the quality of host food resources [36]. For the *Thelebolaceae* in the SA sympatric wildlife group, the discovery has important ecological significance as the proliferation of this specific coprophilous fungal group not only reflects its healthy state [37,38,39], but also suggests its potential key role in nutrient cycling and energy flow. For *Aspergillaceae* in the HA domesticated animal group, the enrichment of *Apiosporaceae* strongly suggests the screening effect of the anthropogenic environment on fungal communities [40]. This discovery is consistent with the characteristics of domesticated animals exposed to stored or processed feed and captive environments, and also suggests that such fungi may serve as potential indicators for evaluating the quality of the breeding environment.

### 4.3. Intra-Group Stability and Variability of Bacteria and Fungi Reflect Ecological Adaptation Mechanisms

The most noteworthy aspect of this study is the significant phenomenon of bacterial community stability and fungal community variability observed in the SA sympatric wildlife group [41]. This significant difference may be attributed to several ecological mechanisms: Firstly, differences in nutrient sources may be the main driving factor [37] as the SA sympatric wildlife usually have a wide diet, and the diversity of their food sources provides rich ecological niches for different types of bacteria, leading to relatively stable bacterial communities [42,43]. However, fungi are more sensitive to environmental changes, and the spread and colonization of their spores largely depend on the stability of microenvironmental conditions such as humidity, temperature, and substrate properties [44]. The SA group animals have a wide range of activities and may be exposed to a more diverse microenvironment, leading to significant differences in fungal communities between individuals. Secondly, variations in host selection pressure could serve as a key mechanism underpinning the tight co-evolutionary linkage observed between bacteria and their hosts, and the host’s immune system and intestinal environment have a strong screening effect on bacterial communities, resulting in relatively consistent bacterial communities within the group [45]. In contrast, the specific interaction between fungi and hosts may be weaker and more affected by the randomness of the external environment, leading to an increase in the variability of fungal communities within the group [46,47]. Marked inter-community variations between bacteria and fungi in the SA group suggest that different microbial groups may have significant differences in their response to ecological factors in natural ecosystems [48,49]. Bacterial communities may be more influenced by host-intrinsic factors such as diet and physiological status, while fungal communities may be more sensitive to external environmental factors such as habitat fragmentation and microclimate condition.

In sharp contrast to the SA group, the HA domesticated animal group showed a high degree of consistency in bacterial and fungal communities, with bacterial and fungal richness ranking first among the four groups. This high degree of consistency reflects the strong selection pressure of the domesticated environment, with unified feeding management, stable feed supply, and relatively single living environment collectively shaping a homogeneous microbial community [50,51,52]. The distribution patterns of bacteria and fungi in the GP and RP groups also showed high consistency. This consistency may be due to the following reasons: First, the filtering effect of specialized diets [53]. Both GP and RP rely on bamboo as their staple food, and this highly specialized diet exerts strong selective pressure on the gut microbiota [54]. Both bacteria and fungi need to adapt to the degradation requirements of bamboo, which is a high fiber, low nutrient substrate, resulting in the relatively consistent distribution pattern of the two microbial communities within the group. On the other hand, the relatively stable habitats may also contribute to this [51,53]. GP and RP usually live in relatively primitive forest environments with less human interference, and their microbial communities are relatively stable [55]. The stability of this environment may affect the community construction of bacteria and fungi at the same time, thus leading to a relatively stable variation pattern within the population. The highly specialized bamboo-feeding behavior of giant pandas is the key factor shaping the microbial community structure of their feces [56]. This behavior is like a filter with the same strength, which makes the bacterial and fungal communities show coordinated and moderate variation within the population. This reflects the unique microecological adaptation mode of giant pandas formed in long-term evolution: maintaining the stability of the core functional microbial community, while still retaining the flexibility to cope with the small differences in food in the natural environment. This discovery marks a major breakthrough in the understanding of the interaction mechanism between host and microorganism, and opens up a new path for innovative wildlife protection strategies.

### 4.4. Limitations and Future Perspectives

While this study provides insights into the fecal microbiota of distinct animal groups, several limitations should be considered. First, the sample size for some groups, especially the sympatric wild animals (SA), remains moderate. Although statistical significance was achieved for key comparisons, larger sample sizes would improve the power to detect subtler differences and enhance the generalizability of the findings. Second, our reliance on amplicon sequencing of the 16S rRNA and ITS genes, while standard, provides taxonomic profiles primarily at the genus level and is subject to well-known technical biases. Metagenomic or metatranscriptomic approaches in future work could offer higher resolution and functional insights. Lastly, despite our efforts to account for major confounding variables, unmeasured environmental factors in the wild may still influence the observed microbial patterns. Acknowledging these constraints is crucial for interpreting our results and guiding future research directions.

While our study identifies microbial patterns across a natural co-habitation gradient, a more targeted experimental design comparing wild and domestic populations would be required to conclusively attribute these patterns to human influence, representing a key avenue for future research. Future studies with balanced designs across taxonomic classes could powerfully disentangle the relative contributions of evolutionary history versus contemporary ecology in shaping gut microbiota in complex animal communities.

## 5. Conclusions

In this study, the fecal microbial communities of giant pandas, red pandas, sympatric wildlife and domestic animals in the Liangshan area were analyzed, and the construction mechanism of microbial communities of wild animals was revealed from the perspective of bacterial–fungal interaction. It is found that the microbial communities of domestic animals are homogeneous under human intervention, while wild animals show more complex ecological adaptation patterns. Innovative findings show that the bacterial community of sympatric wildlife remains stable, while the fungal community shows high variability, revealing the law that the two types of microbial communities are dominated by different ecological factors. More importantly, the study clarified the unique “microecological consistency” strategy of giant panda as a specialized feeding species. Its bacterial and fungal communities showed moderate synergistic variation, reflecting the leading role of feeding screening in the construction of microbial communities. These findings expand the theoretical understanding of host environment adaptation mechanism from the perspective of microbial multi-boundary interaction, and provide a new scientific paradigm for mountain ecosystem protection.

## Data Availability

The original contributions presented in this study are included in the article. Further inquiries can be directed to the corresponding authors.

## Appendix A

**Table A1:** Sample metadata and collection details.

Group	Species (Common Name)	No.of Samples	Collection Location	Freshness at Collection
**GP**	Giant Panda	13	Meigu Dafengding National Nature Reserve (Lanlong, Weiheluo)	3–5 days
**RP**	Red Panda	26	Meigu Dafengding National Nature Reserve (Lanlong, Weiheluo)	2–3 days
**SA**	Tufted Deer	10	Meigu Dafengding National Nature Reserve (Lanlong, Weiheluo)	2–3 days
	Malayan Porcupine	8	Meigu Dafengding National Nature Reserve (Lanlong, Weiheluo)	3–5 days
	Tibetan Macaque	4	Meigu Dafengding National Nature Reserve (Lanlong, Weiheluo)	1–2 days
	Asian Hog Badger	2	Meigu Dafengding National Nature Reserve (Lanlong, Weiheluo)	2–3 days
	Blood Pheasant	2	Meigu Dafengding National Nature Reserve (Lanlong, Weiheluo)	3–5 days
	Lady Amherst’s Pheasant	2	Meigu Dafengding National Nature Reserve (Lanlong, Weiheluo)	2–3 days
**HA**	Domestic Cat	2	Nenghe Village	1 day
	Domestic Dog	5	Nenghe Village	1 day
	Chicken	5	Nenghe Village	1 day
	Domestic Goose	2	Bomalojue	1 day
	Domestic Sheep	9	Bomalojue	1 day
	Domestic Pig	4	Bomalojue	1 day
	Domestic Duck	5	Nenghe Village	1 day
	Domestic Cattle	3	Nenghe Village	1 day
	Domestic Horse	6	Bomalojue	1 day

**Table A2:** Alpha diversity indices of fecal microbiota across different groups.

	GP	RP	SA	HA
		***Bacteria***		
**pd**	18.72 ± 2.15	21.45 ± 1.98	17.23 ± 2.34	23.67 ± 1.87
**Shannon**	3.85 ± 0.42	4.12 ± 0.38	3.68 ± 0.51	4.35 ± 0.35
		***Fungi***		
**pd**	8.42 ± 1.23	9.67 ± 1.45	7.85 ± 1.67	11.23 ± 1.32
**Shannon**	2.15 ± 0.38	2.43 ± 0.41	1.98 ± 0.47	2.68 ± 0.36
